# The Emerging Roles of π Subunit-Containing GABA_A_ Receptors in Different Cancers

**DOI:** 10.7150/ijms.60928

**Published:** 2021-10-31

**Authors:** Iman Imtiyaz Ahmed JUVALE, Zurina HASSAN, Ahmad Tarmizi CHE HAS

**Affiliations:** 1 Department of Neurosciences, School of Medical Sciences, Universiti Sains Malaysia, Health Campus, 16150 Kubang Kerian, Kelantan, Malaysia.; 2 Centre for Drug Research, Universiti Sains Malaysia, 11800 Minden, Penang, Malaysia.

**Keywords:** Cancer, GABA_A_ receptors, π subunit, female reproductive system

## Abstract

Cancer is one of the leading causes of death in both developed and developing countries. Due to its heterogenous nature, it occurs in various regions of the body and often goes undetected until later stages of disease progression. Feasible treatment options are limited because of the invasive nature of cancer and often result in detrimental side-effects and poor survival rates. Therefore, recent studies have attempted to identify aberrant expression levels of previously undiscovered proteins in cancer, with the hope of developing better diagnostic tools and pharmaceutical options. One class of such targets is the π-subunit-containing γ-aminobutyric acid type A receptors. Although these receptors were discovered more than 20 years ago, there is limited information available. They possess atypical functional properties and are expressed in several non-neuronal tissues. Prior studies have highlighted the role of these receptors in the female reproductive system. New research focusing on the higher expression levels of these receptors in ovarian, breast, gastric, cervical, and pancreatic cancers, their physiological function in healthy individuals, and their pro-tumorigenic effects in these cancer types is reviewed here.

## Introduction

Cancer is a broad term used to describe more than 277 different types of diseases (leukemia, melanoma, lymphoma, etc.) that occur in various regions of the body caused by uncontrolled mitotic cell division 1. This uncontrolled growth and division of cells due to genetic mutations results in neoplasms (tumors); therefore, cancer is a (malignant) neoplastic condition. Mutations leading to cancer may arise due to genetic predispositions 2, environmental carcinogens 3, lifestyle choices (e.g., diet, excessive drinking, tobacco smoking, etc.) 4, radiation exposure 5, viral infections 6, 7, and epigenetic changes (e.g., histone modifications, DNA methylation, and microRNA dysregulation) 8. Globally, there were an estimated 19.3 million diagnosed cases and approximately 10.0 million deaths in 2020 related to cancer, excluding nonmelanoma skin cancer 9. Prostate and breast cancer are the most commonly diagnosed cancers in men and women, respectively 10. Notably, a recent 25-year study revealed a significant increase in the mortality rate of patients with breast cancer in both developed and developing countries 11. To curb the cancer mortality rate, current antineoplastic options include chemotherapy 12, radiotherapy 13, immunotherapy 14, hormone therapy 15, surgery 16, precision medicine 17, molecular targeted therapy 18, and stem cell transplantation 19. However, due to the heterogenous and invasive nature of some types of cancer, therapy is not always feasible, leading to poor prognosis and survival rates 20, 21, 22, 23, 24, 25, 26, 27. Therefore, current research is focused on previously undiscovered pathways and other factors (such as receptors) that are involved in cancer development in order to design newer and more effective antineoplastic agents. Recent studies have implicated γ-aminobutyric acid (GABA) type A receptors (GABA_A_Rs), more specifically GABA_A_ receptor containing the π subunit (GABRP), in cancer 28, 29. Therefore, this review highlights the existing knowledge of GABRP expression and function in healthy individuals and its potential role in cancer.

## The GABA_A_ receptors

**Figure 1:**
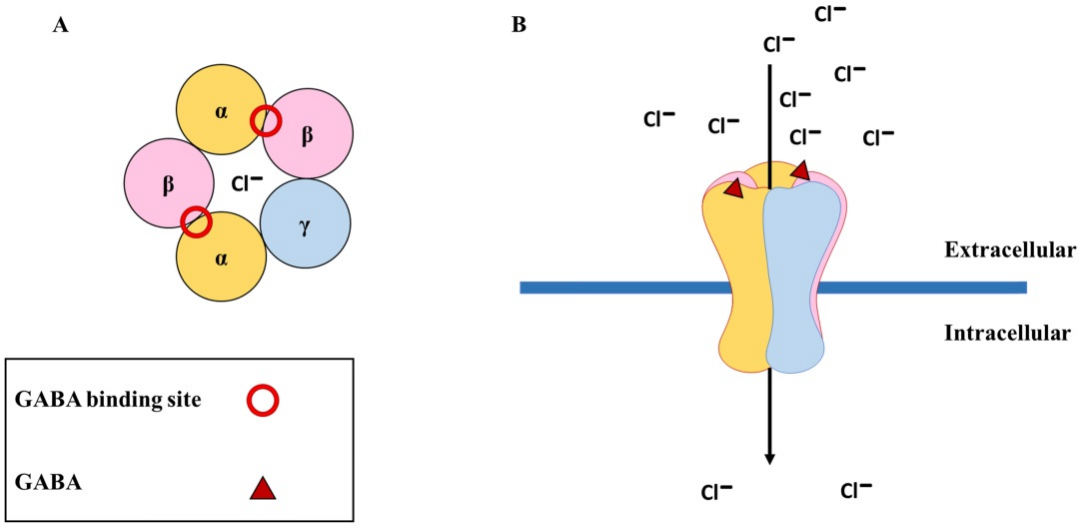
** The physiological function of GABA_A_ receptors.** (**A**) The most common subunit configuration of GABA_A_ receptors along with the binding sites for GABA (shown with red circle); (**B**) The binding of GABA (shown with red triangle) results in the opening of the channel, causing an influx of chloride ions (Cl^-^) into the cell.

**Figure 2:**
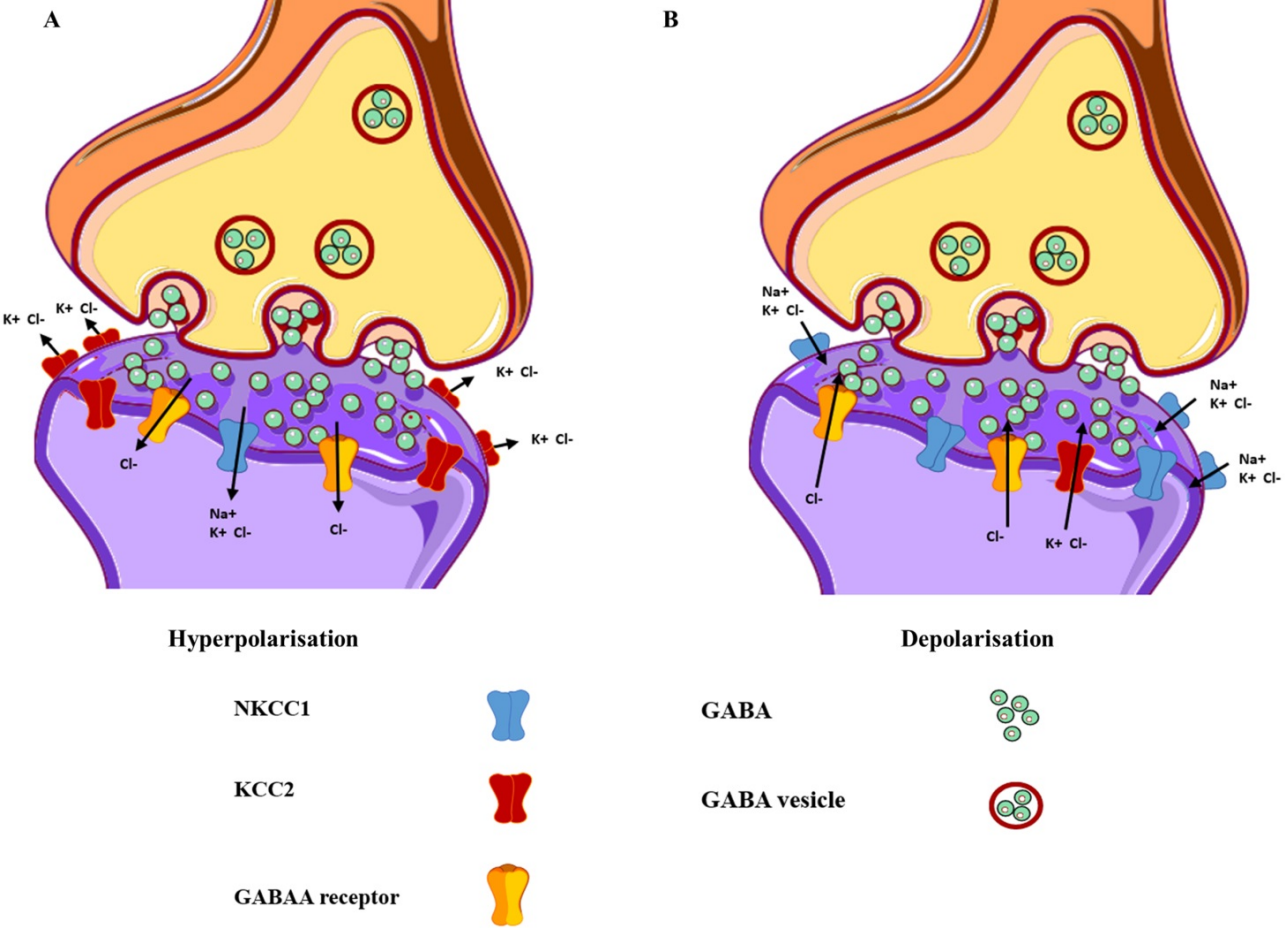
** Differences in the functional and expressional levels of NKCC1 and KCC2.** (**A**) Mature neurons exhibit lower expression levels of NKCC1, as compared to KCC2, resulting in hyperpolarisation. (**B**) Immature neurons express higher levels of NKCC1 in contrast to KCC2, resulting in depolarisation. The yellow structure represents the GABA_A_ receptors, with the green circles representing the neurotransmitter GABA.

GABA is the main inhibitory neurotransmitter of the central nervous system and it generates fast signaling neurotransmissions through the GABA_A_Rs that are cys-loop ligand-gated ion channels comprising five subunits (the main receptor subtype contains two α, two β, and one γ subunit [α-β-α-β-γ]) enclosing a central chloride ion (Cl^-^) pore in the brain 30. To date, 19 subunits have been discovered in the human (α1-α6, β1-β3, γ1-γ3, δ, ε, θ, π, and ρ1-ρ3). When two molecules of GABA bind to the β^+^/α^-^ interface, they cause a conformational change in the receptor structure, resulting in the opening of channels permeable to Cl^-^. This causes an influx of Cl^-^ into the cell, resulting in hyperpolarization of the membrane and inhibition of neuronal signaling 31 (**Figure 1**). When GABA_A_Rs are activated in healthy neurons, hyperpolarization may occur, only if the extracellular concentration of Cl^-^ is greater than the intracellular concentration, resulting in an influx of Cl^-^ into the cell. This Cl^-^ homeostasis is maintained by two essential cation-chloride cotransporters, sodium-potassium-chloride cotransporter isoform 1 (NKCC1) and potassium-chloride cotransporter isoform 2 (KCC2). NKCC1 assimilates Cl^-^ into the cell, whereas KCC2 expels Cl^-^ from the cell 32. In healthy neurons, KCC2 is expressed more than NKCC1, and upon binding of GABA to GABA_A_Rs, there is an influx of Cl^-^ into the cell, which results in hyperpolarization 33 (**Figure 2A**). When the intracellular Cl^-^ concentration exceeds the extracellular concentration, the binding of GABA to the GABA_A_Rs results in an efflux of Cl^-^ from the cell, causing the membrane potential to become more positive, resulting in depolarization 34 (**Figure 2B**). This unique ability of GABA_A_Rs can also be observed in immature neurons during early postnatal development 35. While both the α and β subunits can co-assemble with all other GABA_A_R subunits, the γ and δ subunits cannot coexist within the same receptor subtype 36. Furthermore, only the ρ1, β3, α, and γ subunits can form homo-oligomeric receptors 37. GABA_A_Rs vary in their affinity for GABA, expression sites (synaptic or extrasynaptic), and biophysical and pharmacological properties based on their subunit composition. For example, α subtypes have functional variation; α1 results in sedation, whereas α2 and α3 are instrumental in anxiolysis 38. However, the expression of these subunits is dependent on the other subunits that they co-assemble within the receptor. For instance, the α(1/2/3/5)^+^/γ2^-^ interface is essential for benzodiazepine-mediated sedation, anxiolysis, seizure suppression, and muscle relaxation 38, and only the placement of the α subunit next to the γ subunit (α^+^/γ2^-^) can mediate its function. If both subunits exist within the same receptor but not in the correct order, the resulting GABA_A_R will not be receptive to benzodiazepine. Importantly, not all potential subunit combinations can result in functional GABA_A_Rs. Alterations in receptor composition, such as the switch from a γ2 to a δ subunit could desensitize GABA_A_Rs to drugs such as benzodiazepines 39. Several conditions, such as epilepsy 40, 41, traumatic brain injury 42, 43, mental illnesses (such as schizophrenia and mood disorders) 44, 45, addiction 46, 47, Alzheimer's disease 48, 49, and Parkinson's disease 50, 51, can result in alterations in the subunit composition of GABA_A_Rs, thus highlighting the critical function of subunit configuration in healthy individuals. For more in-depth understanding of the GABA_A_R structure and their ligand-binding site interactions, readers can refer to 30, 52, 53.

## GABRP

### Overview

Although GABRP was first discovered more than 20 years ago, there is not much information currently known about this receptor which possesses atypical functional properties. In comparison to other GABA_A_R subunits, the π subunit is closely related to the β (37%), δ (35%), and ρ (33%) subunits. Previous studies have shown that the π subunit is incapable of forming homo-oligomeric receptors 54. However, it can be co-assembled with α, β, and γ subunits to create αβπ and αβγπ isoforms 55. GABRP exhibits differential pharmacological properties as compared to other similar GABA_A_R isoforms (αβγ, αβδ, and αβε), increased sensitivity to inhibition by zinc ions, no sensitivity to diazepam action, and distinctive neurosteroidal regulation 55. Located on chromosome 5q34, this receptor is expressed in several non-neuronal regions such as the uterus 56, placenta 57, pancreas 29, gastrointestinal tract 58, lungs 59, kidney 60, immune cells 61, and mammary glands 62.

### Female reproductive system

In the uterus, GABRP can alter uterine motility by modulating tissue contractility 54. Its expression levels change throughout pregnancy, with consistent levels throughout gestation followed by a reduction during the onset of labor 63. This change in expression can modify the sensitivity of recombinant receptors to pregnanolone and allopregnanolone 54,63. During gestation, allopregnanolone increases the binding of the GABA_A_R agonist, muscimol, to uterine GABA_A_Rs; in contrast, labor is marked by a limitation in this interaction, which can be attributed to the lower expression levels of GABRP 63. Endometrial levels of GABRP also change during the secretory phase of the uterus, and elevated levels play a crucial role in acquiring endometrial receptivity for embryo implantation 56. Similarly, a recent study found constant placental expression levels of GABRP during gestation, followed by a reduction during labor onset and a complete absence at term 64. This suggests that GABRP has invasive potential and is involved in the development of villous trophoblasts and syncytiotrophoblasts during the first trimester, thus ensuring a secure uterine wall implantation. GABRP can also modulate both anti-apoptotic (B-cell lymphoma 2 [Bcl-2]) and pro-apoptotic (Bcl-2-associated agonist of cell death [Bad] and Bcl-2-like protein 4 [Bax]) protein levels, and elevated placental GABRP levels are implicated in preeclampsia 57, thereby highlighting the pivotal role it plays in the female reproductive system.

### Gastrointestinal tract

In the gastrointestinal tract, GABRP regulates electrolyte transport, with GABA administration resulting in increased intestinal secretion in a dose-dependent manner 58. Increased expression of these receptors has been reported in cases of allergic diarrhea and ulcerative colitis, and treatment with a suitable GABA_A_R antagonist results in alleviation of symptoms 58, 65.

### Lungs

GABRP also plays a role in fetal lung development by governing cell proliferation and/or fluid secretion. During gestation, elevated expression levels of α1, β2, and π subunits have been observed in fetal lung tissue from the initial stage to the later adult stages 59. In one study, fetuses exposed to GABA exhibited a significant increase in body and lung weight with a 30% increase in the total number of saccules, a common marker for lung maturity, as compared to that in a control group. Exposure to GABA also amplified the number of alveolar epithelial type II cells while reducing the amount of α-smooth muscle actin-positive myofibroblasts 59, which indicate conditions such as asthma when present in large numbers 66. Therefore, a reduction in this cell type suggests healthy lung development via GABRP. In another study, epithelial cells exposed to GABA also demonstrated higher Ki-67 levels 59; Ki-67 is another marker for cellular proliferation and the development of healthy lungs, which is absent in resting cells 67. Additionally, GABA regulates Cl^-^ efflux and resolves pulmonary oedema 68. Notably, *GABRP*-knockout studies have reported inhibition of this efflux function 69, further supporting the critical role of GABRP in fetal lung development.

### Kidneys

GABRP has also been detected at both the mRNA and protein levels in human and rat kidneys and may have an autocrine/paracrine mechanism for local GABAergic transmissions 60. Although the receptor composition for GABRP is still debatable due to lack of consistent results from transfection studies, it has been suggested that GABRP in the kidneys is composed of a combination of α1β3π 60.

### Breast

Although several studies have detected the presence of glutamic acid decarboxylase (GAD; the enzyme that synthesizes GABA), GABA 70, and significant expression levels of GABRP in healthy breast tissue, their function remain largely unknown.

## Aberrant GABRP regulation in different types of cancer

### Breast cancer

GABRP expression levels are an important indicator of the risk of recurrence of breast cancer and mortality 62. Almost 50% of all breast cancer types exhibit high GABRP expression levels 71. Elevated levels of GABRP have been previously reported in circulating breast cancer cells 72,73,74 and isolated lymph nodes from patients with breast cancer 75. A multigene real-time reverse transcription polymerase chain reaction (RT-PCR) study observed patients with metastatic breast cancer expressing eight times as much GABRP as compared to stages II-IV patients with no evidence of metastasis 76. This expression level was 30 times higher than that in stage I patients with no evidence of metastasis, suggesting that GABRP expression levels increase with disease progression and metastasis. This elevated expression level of GABRP was also effective in detecting circulating tumor cells in patients with stage I (65%), stages II-IV with no evidence of metastasis (72%), and metastatic (88.5%) breast cancer. Circulating tumor cells serve as an important indicator of the overall survival rate of patients with breast cancer 77. Detection of GABRP expression levels can therefore prove beneficial in tracking disease progression, for instance, when traditional serum markers fail. In one study, most of the healthy controls (51 out of 53) showed low expression levels of GABRP compared to patients with breast cancer 76, and of the 2 remaining controls that exhibited high GABRP levels, 1 participant was pregnant (first trimester). The elevated expression levels of GABRP can be explained by its known physiological role in the female reproductive system. Similarly, *in vitro* studies in basal-like breast cancer (BLBC) cell lines (HCC1187 and HCC70) have also reported elevated expression levels of GABRP 78. Other studies in healthy individuals showed that luminal progenitor breast cells also express high levels of GABRP 79. These cells have been suggested to generate BLBC cells during carcinogenesis, further suggesting a strong connection between GABRP and the BLBC subtype 80. Patients with BLBC often develop secondary cancer in visceral organs such as the lung, liver, and brain when the cancer metastasizes 81,82,83.

Sizemore and colleagues found a strong correlation between GABRP and the formation, migration, and aggressiveness of secondary cancer cells, thus implicating GABRP in brain metastases and poor prognosis. Lentiviral knockdown of *GABRP* in these BLBC cell lines resulted in cytoskeletal alterations, lower expression levels of basal-like cytokeratins (KRT5, KRT6B, KRT14, and KRT17), and reduced phosphorylation of the extracellular signal-regulated kinase (ERK) 1/2 signaling pathway 78. Cytokeratins are structural proteins that form a major component of the intermediate filaments. Since their expression levels vary depending on cell types and their degree of differentiation, cytokeratins serve as suitable markers for differentiating carcinomas from other subtypes of cancer 84. Previous studies have linked GABRP with KRT5, KRT6B, KRT14, and KRT17 in breast cancer pathogenesis 85, as several cytokeratins have been implicated in cancer cell migration 86,87,88. Additionally, cell lines generated with functional GABRP but inhibited ERK 1/2 activity resulted in a lack of this migratory disease phenotype, suggesting that GABRP utilizes the ERK 1/2 signaling pathway to mediate its pro-migratory effects 78. ERK 1/2 is a member of the mitogen-activated protein kinase (MAPK) family and has a highly regulated pathway that plays a crucial role in cell proliferation, differentiation, and stress response. The entire signaling pathway utilizes various kinases, such as Ras/Raf/MAPK-ERK (MEK), ribosomal s6 kinases, MAP kinase-interacting serine/threonine-protein kinases, mitogen- and stress-activated protein kinases, and cytosolic phospholipase A2 89. This pathway is a known modulator of bispecific phosphatases 90,91, subcellular localization of cascade components 92,93, cellular motility 94,95, cytokeratins 78,96,97,98, and other scaffolding proteins 99,100. The ERK 1/2 signaling pathway has been implicated in several cancer subtypes 89,101,102; therefore, abnormal manipulation of this pathway by GABRP can result in carcinogenesis. Triple-negative breast cancer (TNBC) cells have also been reported to primarily express GABRP mRNA and proteins 71. Unlike other types of breast cancer, TNBC cells lack conventional biomarkers such as the oestrogen, progesterone, and human epidermal growth factor receptors and have also been linked to higher rates of relapse and mortality due to its aggressive nature. Therefore, the detection of GABRP mRNA and proteins in these cells could act as potential biomarkers while also providing a site for targeted therapy 103. *In vitro* studies have shown that *GABRP* knockdown inhibits the proliferation of TNBC, whereas *GABRP* silencing suppresses the development of MDA-MB-468 xenografts in nude mice. Moreover, application of anti-GABRP antibodies or *de novo* generated Fabs in TNBC cell lines arrests further cancerous growth. When used in combination with mertansine, similar antineoplastic properties were also observed at nanomolar concentrations 71, further underlining the prospective role of GABRP as a therapeutic target in breast cancer.

### Ovarian cancer

An* in vivo* ovarian cancer study detected a >2-fold increase in the transcriptional expression levels of GABRP in metastatic implants of human ovarian carcinoma xenografts in mice compared to SK-OV-3 ovarian carcinoma cells 104. Another study conducted by the same group found a >4-fold increase in GABRP expression levels in the metastatic tissue of the mice model 28. Utilizing the SK-OV-3 ovarian carcinoma cell lines, several gain-of-function and loss-of-function studies were performed to analyze the role of GABRP in cellular migration and invasion. It was revealed that *GABRP* silencing reduced the invasive and migratory potential of SK-OV-3 cells while downregulating the ERK pathway. Similarly, increased expression of GABRP enhanced cellular invasion and migration and upregulated the ERK pathway. The involvement of GABRP in ERK regulation was further highlighted when the administration of U0126, a MAPK/MEK inhibitor, eliminated the invasive and pro-migratory abilities of SK-OV-3 cells, suggesting that GABRP modulates the MAPK/ERK pathway to enhance the metastatic potential of ovarian cancer 28. Furthermore, a genome-wide DNA methylation profiling study in mouse models detected hypomethylation at the GABRP-963 CpG site. Similar results were also observed in patients who were in the advanced stages of ovarian cancer, implying that the transcriptional regulation of GABRP is governed by a DNA methylation-dependent epigenetic mechanism which further ameliorates the aggressive phenotype of ovarian cancer 28.

### Cervical cancer

Cervical cancer studies have also reported higher expression levels of GABRP in metastatic tissue in patients with cancer as compared to that in controls 105. MicroRNAs are short non-coding RNAs that affect gene silencing by targeting mRNAs at their 3-untranslated region, thus regulating protein expression levels. They are crucial for almost all cellular processes, such as differentiation, development, and homeostasis 106. The microRNA, miR-320c, has been shown to possess anti-tumorigenic properties in cancer development as it downregulates the migratory potential of cancer cells 105. It mediates this function by negatively regulating GABRP protein expression levels in these tissues 105. Rescue studies have shown that patients with reduced expression levels of miR-320c had higher protein expression levels of GABRP, thus developing lymphatic and distant metastases at a higher rate than patients with increased expression levels of miR-320c 105. These high expression levels of GABRP were shown to reverse the effects of miR-320c and increase the migratory potential of cervical cancer cells. Additionally, the upregulation of miR-320c significantly suppressed the migratory potential of HeLa and C33-A cells due to lower expression levels of the GABRP protein. Western blotting studies have indicated that cervical cancer cells that exhibited higher expression levels of miR-320c had significantly lower protein expression levels of GABRP and lower migratory potential 105, implying a possible role for GABRP in metastatic cervical cancer.

### Gastric cancer

*In vitro* studies in KATO III cell lines revealed GABRP-induced proliferative effects in gastric cancer 107. RT-PCR and immunohistochemical studies confirmed that these effects are mediated through a GABA-dependent mechanism in an autocrine or paracrine manner. The integral component of this entire process is the upregulation of the ERK 1/2 pathway via GABRP, which in turn strengthens cyclin D1 expression 107. As previously mentioned, GABA_A_Rs have an inhibitory function (hyperpolarization) based on extracellular Cl^-^ levels. In cancer, there often tends to be a Cl^-^ imbalance that results in depolarization of the membrane, which indirectly activates voltage-gated calcium channels 108. This raises the intracellular calcium ion (Ca^2+^) concentration, which further activates several downstream kinases and signaling pathways. The ERK 1/2 pathway is one such cascade, which upon activation results in the transcriptional upregulation of several genes such as *CCND1* (cyclin D1), which is critical for the progression of the cell cycle from the G1 phase to the S phase 109. Abnormal expression levels of cyclin D1 increase cancer cell proliferation, migration, and metastasis via the Ccnd1·Cdk4-paxillin-Rac1 axis 110. Therefore, irregularities in ERK 1/2 activation due to GABRP can result in cancer. Elevated expression levels of GABRP mRNA and proteins have also been detected in oral squamous cell carcinoma cell lines 111. Additionally, the application of muscimol or GABA further stimulates cellular proliferation, while suppressing apoptosis and arresting the cell cycle in the G2/M phase. Furthermore, when these cells were treated with the GABA_A_R antagonist, S106, and then later re-treated with GABA, they lacked the anti-apoptotic properties that they previously exhibited, strongly supporting the pro-oncogenic nature of GABRP. The modulation of the cell cycle was achieved via GABRP-mediated activation of the p38 pathway and downregulation of the c-Jun N-terminal kinase (JNK) signaling pathway, both of which belong to the MAPK family 111. In healthy cells, activation of the JNK pathway results in the phosphorylation and activation of pro-apoptotic proteins such as Bcl-2-interacting mediator of cell death (BIM; homologous to BAX) and Bcl-2-modifying factor (BMF), which further activates downstream caspases. Simultaneously, JNK can also phosphorylate and inactivate anti-apoptotic proteins, such as death protein 5/harakiri, Bcl-2, and B-cell lymphoma extra-large 112. Therefore, the downregulation of this pathway can result in uncontrolled cell proliferation and can have detrimental effects. In contrast, upregulation of the p38 pathway enhances metastasis and has been correlated with a poor prognosis in cancer 113,114,115.

### Pancreatic cancer

Higher expression levels of GABRP have been observed in all grades of pancreatic ductal adenocarcinoma (PDAC) than in healthy control pancreatic tissues, implying that GABRP plays a critical role in the early stages of pancreatic carcinogenesis 29,116,117. Small interfering RNA-mediated *GABRP* knockdown in PDAC cells was shown to significantly reduce cancer cell proliferation 117. Additionally, the introduction of GABA to these cell lines further increased the growth of GABRP-expressing PDAC cells. However, this was not observed for GABRP-negative cells, implying that GABRP and not any other subtype of GABA_A_Rs are responsible for the tumorigenic phenotype of the PDAC cells. Treatment of GABRP-positive cells with the GABA_A_R inhibitor, picrotoxin, and the calcium channel blocker, nifedipine, restricted cellular proliferation. Moreover, treatment with GABA also increased the intracellular Ca^2+^ levels, which resulted in the activation of the ERK signaling pathway, and picrotoxin or nifedipine could inhibit this activation. Although GABA_A_ receptors cause hyperpolarization in mature neurons, they have been shown to mediate depolarization in immature neurons and glial tumour cells. This activates the voltage gated Ca^2+^ channels causing an increase in intracellular Ca^2+^ levels which results in the phosphorylation and activation of the MAPK/ERK pathway 117. Tissues derived from patients with PDAC have higher levels of GABA due to increased expression levels of GAD1, indicating an autocrine or paracrine-mediated modulation of GABRP in PDAC 117. As previously discussed, aberrant activation of the ERK pathway results in the phosphorylation and activation of several downstream kinases and transcription factors that are essential for cell proliferation, migration, and survival, thus supporting cancerous growth. Another recent study also suggested GABRP-mediated carcinogenesis in PDAC, but in a GABA-independent manner 29. Macrophages are important immune cells that protect the body against harmful microorganisms via phagocytosis, while also serving other essential regulatory and repair functions 119. Moreover, these cells possess the ability to inhibit Th1 cells and the anti-tumor abilities of cytotoxic T lymphocytes, contribute to matrix remodeling, and promote tumor cell invasion and migration 120,121,122. GABRP can govern macrophage infiltration in PDAC cells by coupling with a calcium-activated potassium channel (K_Ca_3.1), which causes an influx of Ca^2+^ and activates the nuclear factor κB. Consequently, this accelerates the expression levels of *CXCL5* and *CCL20* 29, which are known macrophage-recruiting chemokines 123. This results in increased macrophage density which has often been correlated with a poor prognosis in cancer 118. Pharmacological deletion of macrophages by liposomal clodronate greatly reduced the cancer proliferation of GABRP in PDAC cells 29. *GABRP* knockdown reduced the expression levels of chemokines 29, thereby suggesting a unique immunomodulatory role for GABRP in PDAC.

## Conclusion

Although this review summarizes our current knowledge of the GABRP and its enigmatic role in cancer, there are still several areas that require thorough research. A critical component requiring further investigation is its subunit composition, since there is an almost negligible amount of data currently available. Unlike its famous counterparts, the GABRP subunit composition remains shrouded in mystery. GABRP is expressed in several organs and has a few known physiological roles. These recently discovered roles in various cancer subtypes will hopefully direct more attention to these receptors. Although several studies have already highlighted the role of ERK in cancer, there are several factors that could potentially modulate this pathway; GABRP being one of them. GABRP has the potential to serve as a diagnostic marker as well as a possible therapeutic target in cancer. However, further research is needed to better understand these receptors and utilize them as potential targets in cancer therapy.

## Figures and Tables

**Figure 3:**
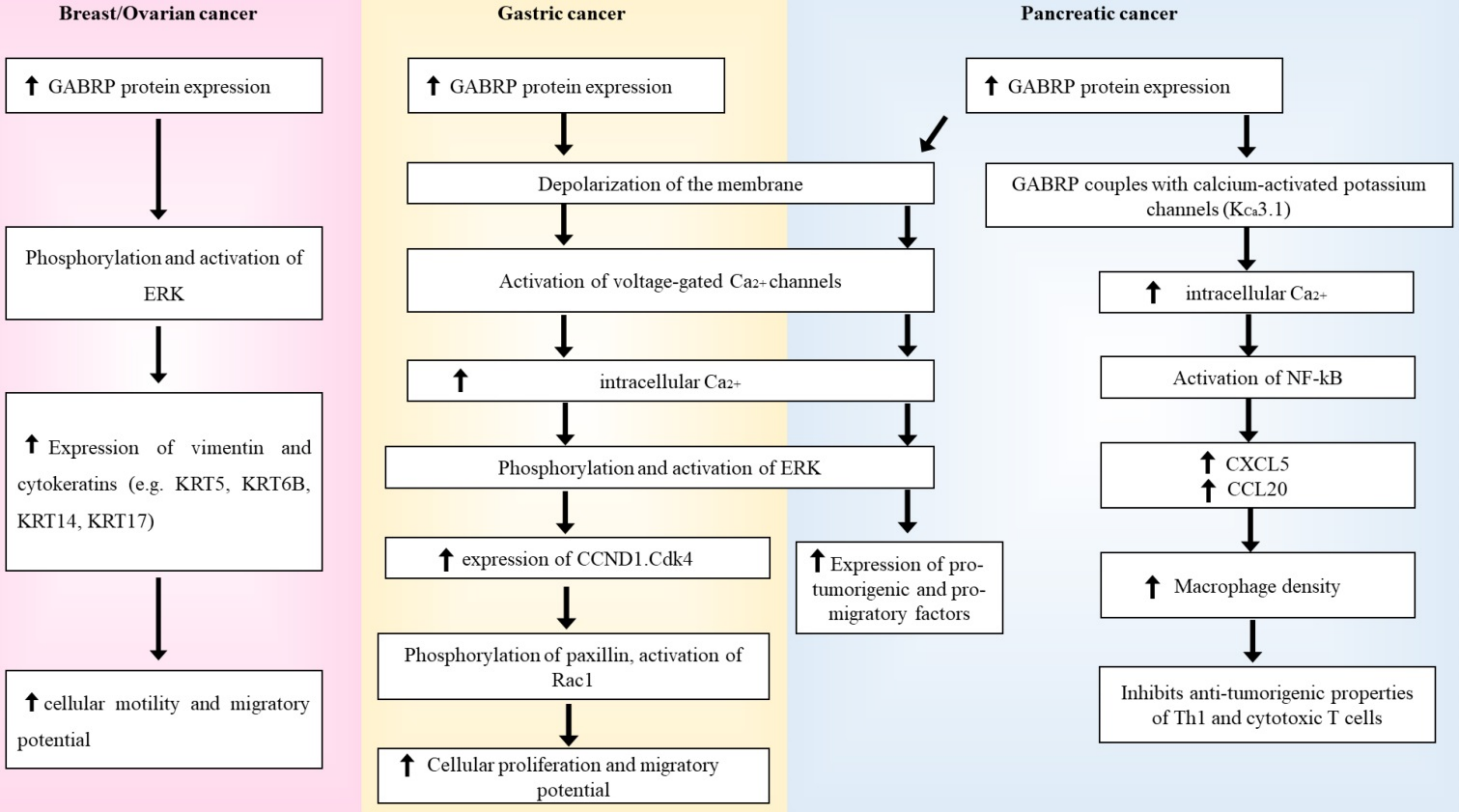
Summary diagram of how GABRP's regulation of ERK in breast/ovarian, gastric and pancreatic cancers.

## Abbreviations

Bcl-2: B-cell lymphoma 2
Bad: Bcl-2-associated agonist of cell death
Bax: Bcl-2-like protein 4
BIM: BCL-2-interacting mediator of cell death
BMF: Bcl-2-modifying factor
BLBC: basal-like breast cancer
ERK: extracellular signal-regulated kinase
GABA: γ-aminobutyric acid
GABAARs: γ-aminobutyric acid type A receptors
GABRP: GABA receptor π subunit
JNK: c-Jun N-terminal kinase
KCC2: potassium-chloride cotransporter isoform 2
MAPK: mitogen-activated protein kinase
NKCC1: sodium-potassium-chloride cotransporter isoform 1
PDAC: pancreatic ductal adenocarcinoma
RT-PCR: real-time reverse transcription polymerase chain reaction
TNBC: Triple-negative breast cancer
